# Are leisure-time and work-related activities associated with low back pain during pregnancy?

**DOI:** 10.1186/s12891-021-04749-w

**Published:** 2021-10-09

**Authors:** Eduardo L. Caputo, Marlos R. Domingues, Andrea D. Bertoldi, Paulo H. Ferreira, Manuela L. Ferreira, Debra Shirley, Marcelo C. da Silva

**Affiliations:** 1 Postgraduate Program in Physical Education, Federal University of Pelotas, Luís de Camões Street, 625, Pelotas, RS 96055-630 Brazil; 2 Postgraduate Program in Epidemiology, Federal University of Pelotas, Pelotas, Brazil; 3 Discipline of Physiotherapy, Faculty of Health Sciences, The University of Sydney, Sydney, NSW Australia; 4 Institute of Bone and Joint Research, The Kolling Institute, The University of Sydney, Sydney, NSW Australia

**Keywords:** Low back pain, Pregnancy, Physical activity, Workload

## Abstract

**Background:**

Although working activities are associated with several pregnancy outcomes, there is scarce information regarding the association between working activities and low back pain (LBP) during pregnancy. This study aimed to investigate whether leisure-time and work-related physical activities during pregnancy are associated with LBP.

**Methods:**

Data from the 2015 Pelotas Birth Cohort study were used. Demographic, socioeconomic, gestational, leisure-time (prior to and during pregnancy) and work-related (days of work, hours of work, standing and heavy lifting) physical activity data were collected at birth. LPB was assessed in the 12-month follow-up period.

**Results:**

Leisure-time physical activity either prior to and during pregnancy was not associated with LBP. Working during pregnancy, days of work and standing position at work were not associated with self-reported LBP during pregnancy. However, working more than 8 h per day and always lifting heavy objects at work increased the odds ratio for LBP (OR 1.30 95%CI: 1.04; 1.63; and OR: 1.39 95%CI 1.08; 1.81, respectively). In addition, women who had lifted heavy objects often/always, reported an increase in pain intensity.

**Conclusion:**

Working during pregnancy and days worked per week were not related to experiencing LBP. However, women who worked more than 8 h per day, as well as women who lifted heavy objects at work on a regular basis, were more likely to experience pregnancy-related LBP.

**Supplementary Information:**

The online version contains supplementary material available at 10.1186/s12891-021-04749-w.

## Introduction

Low back pain (LBP) is a disabling condition with a global prevalence of approximately 40% [1]. Women are more likely to experience LBP, especially during pregnancy. The prevalence of pregnancy-related LBP is estimated to be above 50%, representing an important health problem related to pregnancy [1, 2].

Physically demanding jobs and leisure-time physical activity may have opposite effects on health. Higher levels of occupational physical activity increase the risk of absence due to sickness by 84%, whilst leisure-time physical activity decreases the risk by 23% [3]. This contrasting effect of leisure-time and occupational activity is also observed in LBP. Work activity increases the risk of LBP by 27% and 24% in women and men, respectively, whilst leisure-time physical activity decreases the risk in general population by 16% [4, 5].

Structured exercise interventions have been shown to reduce the risk of LBP during pregnancy by 9% [6]. However, intervention studies generally include small sample sizes (ranging from *n* = 42 to *n* = 257) and are often focused on specific exercise training protocols, such as resistance or strength training, making it difficult to develop inferences at population level [6]. Only one study conducted in Norway focused on an association between experiencing LBP and physical activity level during pregnancy, measured as exercise frequency (e.g. days per week) in a large sample of pregnant women, and found that women who engaged in exercise twice per week were less likely to report LBP [7].

Working hours, heavy lifting, prolonged standing, highly repetitive work and heavy physical work are the most common occupational exposures [8, 9]. However, there is weak evidence showing a dose-response effect of standing or walking, and LBP, as well as conflicting evidence between occupational lifting and LBP in working populations [10, 11].

Working activities during pregnancy (e.g. prolonged time standing or walking, squatting or kneeling) have been studied to understand their influence on outcomes such as preterm delivery, low birth weight and pre-eclampsia [9, 12]. Furthermore, there is scarce information regarding the association between working activities and LBP in pregnant women [13, 14] . The aim of this study was to investigate whether leisure-time (prior to and during pregnancy) and paid work-related physical activities during pregnancy (work during pregnancy, days of work, hours of work, standing and heavy lifting) are related to LBP.

## Methods

### Study design and participants

Data from the 2015 Pelotas Birth Cohort were used. This cohort was developed to evaluate long-term maternal-child outcomes through longitudinal data collection. All maternity hospitals in the city of Pelotas, Brazil were included in the study. Four of the five maternity hospitals in the city were assessed by on-site interviewers, and an interviewer visited the remaining hospital on a daily basis to assess births. This strategy allowed us to assess all births in Pelotas between January 1st and December 31st, 2015. All participants signed an informed consent to take part in the study, and if they were under 18, a legal guardian consent was obtained. More details on the methods of this cohort study are available elsewhere [15].

A cross-sectional study nested in the 2015 Pelotas Birth Cohort Study was conducted. Demographics, behavioral, gestational, and work-related data were collected at perinatal assessment, when women were interviewed within 24 h after delivery using a questionnaire. LBP during pregnancy was collected at the 12-month assessment following delivery, when women were interviewed at their location of preference (e.g. at home, work) [16].

### LBP

Women were asked about LBP experience during pregnancy through the following question: *“Have you experienced low back pain during pregnancy?”*. Also, a numeric pain rating scale was used to assess pain intensity during pregnancy, where “0” indicated no pain and “10” the highest pain level.

### Covariates

Sociodemographic, behavioral and gestational characteristics were used as covariates. Age was defined in complete years and then divided into four categories: 13–19, 20–29, 30–39 and 40–47 years. Family income was categorized into quintiles. Education level was assessed as complete years of formal education, and divided into four categories: 0–4, 5–8, 9–11, and ≥ 12 years. Behavioral characteristics (smoking), birth type (caesarean or vaginal), health-related problems (high blood pressure, eclampsia, depression, and urinary infection) and pregnancy characteristics (parity, gestational weight gain, and multiple births) were also assessed.

### Leisure-time physical activity

Time, frequency, and duration of leisure-time physical activity were assessed using the following questions: “*Which physical activities did you engage in this period?*”; “*How many times a week*?”; and “*How long did each session usually take*?”. These questions were based on their usual routines 3 months before and during first, second and third trimester of pregnancy. Total amount of leisure-time physical activity was estimated by the sum of minutes per week spent on each activity in each trimester of pregnancy. The sum of the three trimesters was used to estimate the total amount of physical activity during pregnancy. The instrument has been previously used in similar studies [17]. Based on the weekly 150-min physical activity recommendation, participants were classified as active (≥ 150 min/week) or inactive (< 150 min/week) [18, 19].

### Physical activity status during pregnancy

A status of physical activity was created based on physical activities performed in each trimester of pregnancy. Women were classified into four different categories: i) did not engage in recommended levels of physical activity (*never*); ii) engaged only for one trimester (*at least 1 tri*); iii) for two trimesters (*at least 2 tri*); iv) all pregnancy periods (*always*) [16].

### Work-related activity

Women were asked about paid work during pregnancy with the question: “*Did you work during pregnancy?*”. Total number of days and hours worked per day during pregnancy were obtained with the following questions: “*During pregnancy, how many days per week did you work*?” and “*During the working days, how many hours per day did you work*?”. For analyzes purposes these variables were categorized as: days (up to 5 days per week / more than 5 days per week), and hours (up to 8 h daily / more than 8 h daily).

Standing and heavy lifting at work were recorded. Standing at work was assessed through the question: “*During your work routine how much time did you spend in standing position*?”. Heavy lifting at work was assessed by the question “*Did you have to lift heavy items at work*?”. There were five possible answers: “*never”, “rarely, “sometimes”, “often”* and “*always*”.

### Statistical analysis

A group of 142 pregnant women who were randomly enrolled in an intervention group of a randomized controlled trial nested in the cohort study were excluded from analyzes. This original trial studied the effects of an exercise program during pregnancy on mother and child health outcomes [20]. Descriptive data were presented as total frequencies and percentages. One-way ANOVA and independent t-tests were used to analyze differences of pain intensity and levels of leisure-time physical activity. A hierarchical model of analyzes with two levels was conducted (1st level: age, income and education level; 2nd level: eclampsia, depression, urinary infection, smoking, weight gain, parity and multiple births). Logistic regression models were used to analyze the relationship between leisure-time and work-related physical activities and self-reported LBP. Odds ratios (OR) and 95% confidence intervals (95%CI) were obtained from logistic regression models (LBP). Similarly, β coefficients and 95%CI were obtained from linear regression models (pain intensity). Variables that presented a *p*-value <.2 in univariable analyzes were retained in the multivariable models. A p-value of <.05 was assumed for statistical significance. All analyzes were conducted using STATA statistical software (StataCorp. 2015, Stata Statistical Software: Release 14, Version 14.0, StataCorp LP, College Station, TX, USA).

## Results

### Sample characteristics

We used data from 3827 women. The prevalence of LBP during pregnancy was 41.9%. Sample characteristics are summarized in Table 1. Most women were in the 20–29 age group (47.1%), attended 9–11 years of formal education (35.1%), and did not smoke (84.1%). During pregnancy, most women did not present eclampsia (93.5%), depression (88.4%) or urinary infection (54,4%).

**Table 1:** Sociodemographic and pregnancy-related characteristics of women who provided self-reported information about LBP during pregnancy in the 2015 Birth cohort. (*n* = 3827)

*Age (years)*	N (%)
13–19	562 (14.7)
20–29	1802 (47.1)
30–39	1349 (35.3)
40–47	113 (2.9)
*Schooling (years)*	
0–4	332 (8.7)
5–8	989 (25.9)
9–11	1343 (35.1)
≥ 12	1161 (30.3)
*Family Income*	
1 (poorest)	761 (19.9)
2	764 (19.9)
3	771 (20.3)
4	782 (20.4)
5 (wealthiest)	747 (19.5)
*Smoking*	
No	3216 (84.1)
Yes	608 (15.9)
*Type of birth*	
Vaginal	1352 (35.3)
Caesarean	2474 (64.7)
*Eclampsia*	
No	3568 (93.5)
Yes	246 (6.5)
*Depression*	
No	3380 (88.4)
Yes	445 (11.6)
*Urinary infection*	
No	2079 (54.4)
Yes	1740 (45.6)
*Multiple birth*	
No	3728 (97.4)
Yes	99 (2.6)
*Parity*	
1	1684 (44.0)
2	1169 (30.6)
3 or more	972 (25.4)

Participants reporting LBP were less engaged in physical activities both before and during pregnancy. Furthermore, participants with LBP worked less (days per week) and reported a lower workload (hours per day) during pregnancy. Nevertheless, longer hours of standing and a high frequency of lifting heavy items were reported by women who experienced LBP during pregnancy. On the other hand, a higher frequency of lifting heavy items was reported by women experiencing LBP during pregnancy (Supplemental Material).

### LBP and physical activity

**Fig. 1:**
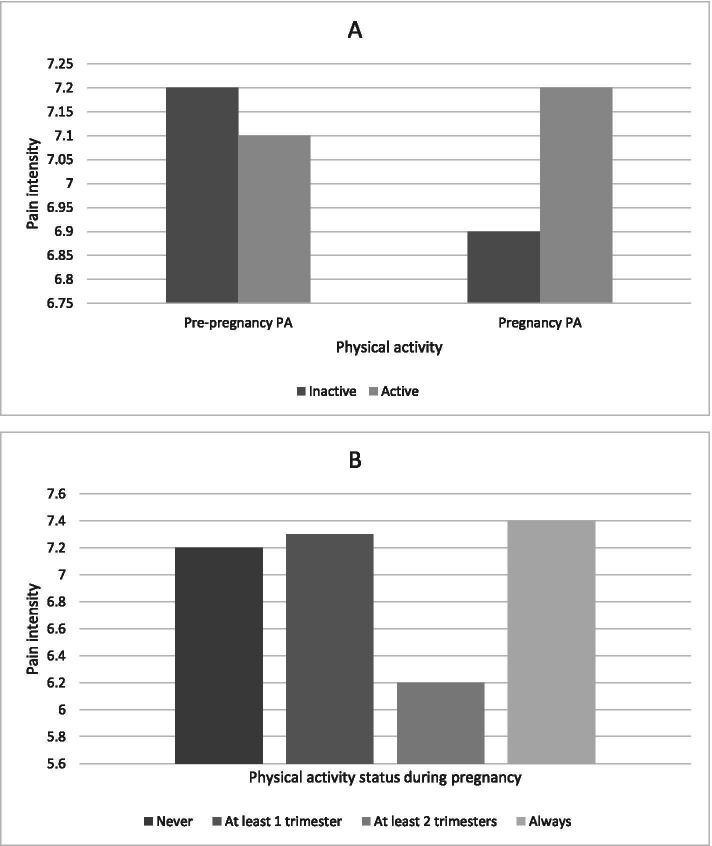
**A** Physical activity pre- and during pregnancy and pain intensity. **B** Physical activity status and pain intensity.

No differences were observed between pain intensity levels and pre- and during pregnancy physical activity (Fig. 1). In the same way, there was no association between LBP and leisure-time physical activity either before (OR: 0.99 95%CI: 0.82 to 1.19) or during pregnancy (OR: 0.97 95%CI: 0.77 to 1.21), as well as physical activity status during pregnancy (Table 2).

**Table 2:** Associations between self-reported LBP during pregnancy and leisure-time physical activity before and during pregnancy. (N=3746))

	Self-reported LBP during pregnancy			
	Univariate		Multivariate	
	OR (95% CI)	p-value	OR (95% CI)	***P***-value
*Prepregnancy PA* ^£^	1.06 (0.88 to 1.26)	0.514	0.99 (0.82 to 1.19)	0.960
*Pregnancy PA* ^£^	1.01 (0.81 to 1.26)	0.892	0.97 (0.77 to 1.21)	0.817
*PA status* ^£^		0.344		0.262
Never	–		–	
At least 1 trimester	1.21 (0.86 to 1.71)		1.14 (0.80 to 1.62)	
At least 2 trimesters	0.75 (0.46 to 1.22)		0.72 (0.43 to 1.19)	
Always	0.81 (0.47 to 1.39)		0.81 (0.47 to 1.39)	

£ adjusted for age, family income, depression, urinary infection, gestational weight gain, parity and multiple pregnancy

*Note*: Prepregnancy PA, pregnancy PA, and PA status are referring to participants performing ≥150 min of physical activity per week

### Self-reported LBP and work activity

Working during pregnancy was associated with self-reported LBP in univariable but not in multivariable analyzes (OR:1.10 95% CI:0.96 to 1.28). In the same way, pain intensity was associated with working days and mean standing hours at work in univariable but not in multivariable analyzes (β 0.27 95%CI -0.41; 0.58; β 0.05 95%CI -0.003; 0.10, respectively) (Table 3).

**Table 3:** Associations between self-reported LBP during pregnancy and work-related activities

	Univariate		Multivariate		Univariate		Multivariate	
	OR (95% CI)	***P***-value	OR (95% CI)	***P***-value	β (95% CI)	***P***-value	β (95% CI)	***P***-value
*Working during pregnancy* ^€^ (*n = 3748*)		0.002		0.166		< 0.001		0.189
No	1.00		1.00		Ref		Ref	
Yes	1.23 (1.08; 1.40)		1.10 (0.96; 1.28)		−0.02 (−0.03; −0.01)		−0.17 (−0.43; 0.09)	
*Days of work* ^€^ (*n = 2123*)		0.537		0.566		0.011		0.089
Up to 5 days per week	1.00		1.00		Ref		Ref	
More than 5 days per week	0.94 (0.80; 1.12)		0.95 (0.79; 1.14)		0.40 (0.09; 0.71)		0.27 (−0.41; 0.58)	
*Hours of work* ^€^ (*n = 2114*)		0.041		0.023		0.044		0.049
Up to 8 h daily	1.00		1.00		Ref		Ref	
More than 8 h daily	1.26 (1.01; 1.58)		1.30 (1.04; 1.63)		0.39 (0.01; 0.79)		0.39 (0.001; 0.77)	
		0.192		0.127		0.031		0.066
*Mean standing hours work* ^€^ (*n = 2131*)	1.02 (0.99; 1.05)		1.03 (0.99; 1.06)		0.06 (0.01; 1.11)		0.05 (−0.003; 0.10)	
*Heavy lifting* ^€^ (*n = 2114*)		0.015		0.011		< 0.001		0.001
Never	1.00		1.00		Ref		Ref	
Rarely	0.84 (0.55; 1.28)		0.83 (0.54; 1.27)		0.51 (−0.28; 1.30)		0.53 (− 0.25; 1.31)	
Sometimes	1.19 (0.93; 1.53)		1.20 (0.93; 1.55)		−0.09 (− 0.63; 0.35)		− 0.19; (− 0.63; 0.25)	
Often	1.33 (0.92; 1.94)		1.37 (0.94; 2.00)		0.86 (0.23; 1.49)		0.81 (0.18; 1.44)	
Always	1.34 (0.98; 1.13)		1.39 (1.01; 1.93)		1.13 (0.60; 1.66)		0.92 (0.39; 1.44)	

^€^ adjusted for age, family income, depression, urinary infection, gestational weight gain, parity and multiple pregnancy

Working more than 8 h daily was associated with both self-reported LBP (OR: 1.30 95%CI:1.04 to 1.63) and increased pain intensity (β 0.39 95%CI 0.001; 0.77). Always lifting heavy items at work (OR: 1.39 95%CI 1.01 to 1.93) was associated with self-reported LBP during pregnancy. Furthermore, an increase of almost one point on the numerical pain scale was observed in women who reported lifting heavy items often (β 0.81 95%CI 0.18; 1.44), and always (β 0.92 95%CI 0.39; 1.44) (Table 3).

## Discussion

### Summary of findings

Leisure-time physical activity either before or during pregnancy, as well as physical activity status were not associated with LBP during pregnancy. Regarding work-related activities, working during pregnancy and days of work were not associated with LBP during pregnancy, either. However, women who worked more than 8 h per day, as well as women who had to lift heavy items frequently at work experienced LBP, as well as reported increased pain levels during pregnancy.

### LBP and leisure-time physical activity

Data from randomized controlled trials showed that structured exercise programs can decrease the risk of experiencing LBP during pregnancy [6]. A study conducted in Norway reported that exercising twice a week during pregnancy was associated with a 20% reduced odds ratio of LBP during pregnancy (OR 0.80 95%CI 0.66 to 0.97) [7]. However, a dose-response relationship between LBP and exercise frequency could not be detected, as there was no association between LBP and exercising three or more times a week (OR 0.82 95%CI 0.68 to 1.02) [7].

Although literature indicates a positive effect of structure exercise programs in LBP during pregnancy, there is still a lack of evidence regarding the relationship of leisure-time physical activity and this outcome [6]. Our aim assessing physical activity status during pregnancy was to understand whether being active in specific periods or throughout the entire pregnancy could change LBP occurrence. During pregnancy, most women decrease leisure-time physical activities, evidenced by a linear trend of discontinuation from 1st to 3rd trimesters of pregnancy [17, 21]. This relationship is inversely proportional to LBP during pregnancy, as the likelihood of experiencing LBP increases from the beginning of pregnancy until birth [13, 22]. LBP was not assessed by pregnancy period (first, second and third trimesters), such as physical activity. This might explain the lack of association between leisure-time physical activity and LBP during pregnancy observed in our findings.

### LBP and work-related activity

The existing literature regarding work-related activities and gestational outcomes aimed to evaluate the relationship of work load and low birth weight, preterm delivery, miscarriage and small head circumference [9, 23]. However, there is a lack of evidence regarding LBP and work-related activities in pregnant women. Hormonal, vascular, biomechanical, behavior, and physical factors, among others are associated with pregnancy-related LBP [24–26].. Although we have not found an association between LBP and days of work, a significant association was observed for daily working hours and LBP during pregnancy. Previous evidence indicates that low academic levels are associated with pregnancy related-LBP [25]. Women with high academic level (e.g. University Degree) might work less hours, as well as have a less extraneous work, which may explain our findings regarding daily work load [13].

Previous studies have found that prolonged standing and heavy lifting are the most common work-related activities associated with LBP in women [4, 27]. However, there is no clear causal relationship to explain the effect of occupational standing and LBP in working age population. Because of spinal mechanical changes during pregnancy, related to a shift in center of gravity, standing may increase pregnant women’s chance of experiencing LBP [24]. Also, this relationship could be modified by time spent on the activity, environmental conditions, and postural/body-weight issues [11]. However, our data is in agreement with a previous study which did not find association between the Oswestry score for back pain and standing at work at 20 and 34 weeks of pregnancy [14]. Further studies are necessary to better understand this relationship.

Occupations that include standing, walking and heavy lifting increase the risk of women experiencing LBP by 27% [4]. It has been shown that intensity (load) and frequency (days per week) of lifting have an exposure-response relationship with LBP incidence (OR 1.09 95%CI 1.03 to 1.15 and OR 1.11 95%CI 1.05 to 1.18, respectively) [23]. Although detailed information on frequency and intensity of heavy lifting was not available, we observed that women who reported lifting heavy items during pregnancy on a regular basis were more likely to experience LBP, as well as higher pain levels.

### Strengths and limitations

Some limitations of the present study should be addressed. The cross-sectional design of our study, concerning physical activity during pregnancy and self-reported LBP could lead to a reverse causality bias. As LBP was assessed retrospectively, at 12-month assessment, recall bias could be expected as well. Also, as physical activity was assessed by self-report, over-(or under-) estimation cannot be discarded [28]. Finally, studies involving working activities could be influenced by the healthy worker effect (bias), since women could be away from work due to issues related to pregnancy [29].

Studies have investigated the association between work-related activities and pregnancy outcomes (e.g. preterm delivery, low birth weight) [9, 12, 30]. To the author’s knowledge the present study is the first to investigate an association between work-related activities and LBP in pregnant women. Additionally, our study is based on a representative sample of pregnant women, as every woman who gave birth in 2015 in Pelotas were included.

## Conclusion

No relationship was observed between LBP during pregnancy and leisure-time physical activity, both pre and during pregnancy. In the same way, no relationship was found between LBP during pregnancy and work (including long hours of standing) in this study. However, women working more than 8 h a day and/or lifting heavy items frequently and on a regular basis were more likely to report pregnancy-related LBP. Further longitudinal studies during pregnancy, with data collection throughout gestation, are necessary to understand the causal relationship between working activities and LBP in pregnant women.

## Supplementary Information

**Additional file 1.**

## Data Availability

The data that support the findings of this study are available from Center of Epidemiology Research (Federal University of Pelotas), but restrictions apply to the availability of these data, which were used under license for the current study, and so are not publicly available. Data are however available from the authors upon reasonable request and with permission of Cohorts commission of the Center of Epidemiology Research (http://www.epidemio-ufpel.org.br/site/content/home/index.php).
