# Supplementary material for: Are leisure-time and work-related activities associated with low back pain during pregnancy?
Source: BMC Musculoskelet Disord. 2021 Oct 9;22:864. doi: 10.1186/s12891-021-04749-w (PMC8502401; doi:10.1186/s12891-021-04749-w)
Supplement: Supplementary file 1 — Additional file 1. [file 12891_2021_4749_MOESM1_ESM.docx]

| **Supplemental Material.** LBP during pregnancy and work-related and leisure-time physical activities among women from the 2015 Pelotas birth cohort. | | |
| --- | --- | --- |
|  | **Self-reported LBP during pregnancy** | |
| *Characteristics* | **No** | **Yes** |
|  |  |  |
| *Previous PA* |  | |
| No | 1,884 (58.3) | 1,348 (41.7) |
| Yes | 336 (56.9) | 255 (43.1) |
|  |  |  |
| *Pregnancy PA* |  |  |
| No | 2,011 (58.1) | 1,450 (41.9) |
| Yes | 209 (57.7) | 153 (42.3) |
|  |  |  |
| *Working during pregnancy* |  | |
| No | 1,016 (60.9) | 651 (39.1) |
| Yes | 1,207 (55.9) | 952 (44.1) |
|  |  | |
| *Days of work* |  |  |
| Up to 5 days per week | 654 (55.2) | 530 (44.8) |
| More than 5 days per week | 547 (56.6) | 420 (43.4) |
|  |  |  |
| *Hours of work* |  |  |
| Up to 8 hours daily | 1,007 (57.0) | 760 (43.0) |
| More than 8 hours daily | 192 (51.2) | 183 (48.8) |
|  |  |  |
| *Mean standing hours work** | 3.0 ± 2.7 | 3.2 ± 2.9 |
|  |  |  |
| *Heavy lifting* |  |  |
| Never | 834 (69.7) | 623 (68.0) |
| Rarely | 53 (4.9) | 96 (4.5) |
| Sometimes | 154 (12.9) | 291 (13.6) |
| Often | 60 (5.0) | 120 (5.6) |
| Always | 89 (7.4) | 178 (8.3) |
| PA = Physical activity  *Mean ± SD |  |  |
